# Nogo-A Drives Alzheimer’s Disease Progression by Inducing Tauopathy Vulnerability

**DOI:** 10.14336/AD.2024.0053

**Published:** 2024-04-23

**Authors:** Zijian Wang, Jun-ping Pan, Jiayuan Geng, Shijie Lv, Guisi Chen, Nian Fang, Zheng Zhang, Junliang Li, Xinke Xu, Rui Wang, Qing Zheng, Li Yan, Guobing Chen, Fei Xiao

**Affiliations:** ^1^Department of Pharmacology, School of Medicine, Jinan University, Guangzhou, China.; ^2^Department of Microbiology and Immunology, Institute of Geriatric Immunology, School of Medicine, Jinan University, Guangzhou, China.; ^3^Guangdong-Hong Kong-Macau Great Bay Area Geroscience Joint Laboratory, Guangzhou, China.; ^4^Guangdong Second Provincial General Hospital, Postdoctoral Research Station of Basic Medicine, School of Medicine, Jinan University, Guangzhou, China.; ^5^Department of Microbiology and Biochemical Pharmacy, School of Pharmacy, Jinan University, Guangzhou, China.; ^6^Department of Pharmacy, The First Affiliated Hospital of Jinan University, Guangzhou, China.; ^7^Department of Neurosurgery, Guangzhou Women and Children's Medical Center, Guangzhou, China.; ^8^Department of Radiology, Guangdong Provincial People's Hospital, Southern Medical University, Guangzhou, China.; ^9^Formula-Pattern Research Center, School of Traditional Chinese Medicine, Jinan University, Guangzhou, China.; ^10^Key Laboratory of Viral Pathogenesis & Infection Prevention and Control (Jinan University), Ministry of Education, Guangzhou, China

**Keywords:** Nogo-A, Tau hyperphosphorylation, Alzheimer's disease

## Abstract

Tauopathies, a group of neurodegenerative disorders, are characterized by disrupted homeostasis of the microtubule binding protein tau. Nogo-A mainly hinders axonal growth and development in neurons, but the underlying mechanism of tau vulnerability has not been determined. Here, to gain more comprehensive insights into the impact of Nogo-A on tau protein expression, we showed that Nogo-A induces tau hyperphosphorylation, synapse loss and cognitive dysfunction. Consistent with the biological function of tau hyperphosphorylation, Nogo-A-induced tau hyperphosphorylation altered microtubule stability, which causes synaptic dysfunction. Mechanistically, Nogo-A-induced tau hyperphosphorylation was abolished by the Nogo-A antagonist NEP1-40 in primary neurons. Surprisingly, downregulation of Nogo-A in the hippocampus of AD mice (hTau. P301S) inhibited tau hyperphosphorylation at the AT8, Thr181, The231 and Ser404 sites and rescued synaptic loss and cognitive impairment in AD mice. Our findings exhibit a strong degree of consistency with Nogo-A-induced tauopathy vulnerability, reinforcing the coherence and reliability of our research. Furthermore, in mice, Nogo-A increases tauopathy vulnerability to exacerbate AD progression via ROCK/AKT/GSK3β signaling. Together, our findings provide new insight into the function of Nogo-A in regulating tau hyperphosphorylation and reveal an effective treatment strategy for tauopathies.

## INTRODUCTION

Neurofibrillary tangles (NFTs) are composed of hyperphosphorylated tau aggregates and are the pathological hallmarks of tauopathies such as Alzheimer's disease (AD). In 1907, Alois Alzheimer first described NFTs as some of the major brain lesions in AD [1]. However, it was not until 1985 that the major component of NFTs was identified as hyperphosphorylated tau [2, 3]. It has been demonstrated that tau phosphorylation at disease-related sites reduces the ability of tau to bind to microtubules, resulting in loss of tau function, suggesting that tau phosphorylation plays a critical role in tauopathies [4, 5].

AD is the most common form of dementia and was first described by the German psychiatrist/ neuropathologist Alois Alzheimer in 1906 [6]. Although the mechanisms underlying AD pathogenesis are unclear, multiple findings support two main hypotheses: the β-amyloid hypothesis and the tau hypothesis. The β-amyloid hypothesis proposes that AD is caused by the deposition of β-amyloid (amyloid beta protein, Aβ) [7], and the tau hypothesis suggests that AD results from hyper-phosphorylated tau in NFTs. However, Aβ-targeted therapies have been shown to be minimally effective in clinical trials, shifting focus to the tau hypothesis of AD. Studies have shown that the number of NFTs, not the number of Aβ plaques, is positively correlated with the severity of dementia in AD patients [8]. Therefore, therapies targeting tau hyperphosphorylation have received great attention for the treatment of AD [9].

Nogo-A or reticulon-4A (RTN4A), the largest variant of Nogo, is a myelin protein encoded by the Nogo gene Reticulon4 (RTN4). Nogo-A is predominantly expressed in oligodendrocytes and plays a crucial role in neuronal regeneration. Fragment analysis and binding studies revealed that Nogo-A contains multiple functional domains. Among them, the Nogo-66 loop and Nogo-A-Δ20 have been shown to inhibit neurite outgrowth. In the past decade, Nogo-A expression has been reported to be increased in the brains of AD patients [10-12]. However, the effect of Nogo-A on the AD process is still unclear. The present study hypothesized that Nogo-A may be involved in AD progression by driving tauopathy. Therefore, the aim of this study was to investigate whether Nogo-A affects tau phosphorylation, tau pathology and cognitive function and the underlying mechanism in vitro and in vivo. Furthermore, this study explored the possible therapeutic effect of downregulating Nogo-A expression in aging transgenic tau mice. The present study provides new information about Nogo-A function and an effective therapeutic target for tauopathies.

## MATERIALS AND METHODS

### Animals and treatment

C57BL/6 mice were purchased from Guangzhou Yancheng Biotechnology Research Institute. The Thy1-hTau.P301SCBA.C57BL/6 (hTau. P301S) mice were donated by Prof. Michel Goedert, University of Cambridge, under MTA agreement [13]. The mice were housed in the SPF animal room of the Experimental Animal Center of Jinan University, which complies with the EU Directive 2010/63/EU guidelines for animal experiments. The environmental conditions included a controlled temperature (20 ± 2 °C), moderate humidity (50-60%), and a 12/12 h light/dark cycle. All experiments were conducted using protocols approved by the Animal Research Committee of the School of Medicine of Jinan University (approval no. IACUC-20190319-010).

### Primary neuron culture

Sprague-Dawley (SD) rats were purchased from Guangdong Provincial Medical Laboratory Animal Center. Primary neuron cultures were prepared from postnatal day 0 (P0) rat pups. The mouse brain was carefully removed and immersed in D-Hank's solution (Thermo Fisher). Subsequently, the cerebral cortex was isolated and minced using scissors for 1 minute, followed by dissociation into a single-cell suspension through 0.125% trypsin (Thermo Fisher) digestion for 20 minutes. The digestion process was stopped by adding DMEM/F12 (Thermo Fisher) supplemented with 10% (v/v) fetal bovine serum (FBS) (Thermo Fisher). After dissociation and passage through a 75-μm nylon cell strainer, the cells were centrifuged at 1000 rpm for 3 minutes, resuspended at the desired density, and seeded on glass coverslips coated with poly-D-lysine (Thermo Fisher). After 4 hours, the medium was replaced with neurobasal medium containing 2% B27 (Thermo Fisher) and 1% penicillin/streptomycin (Thermo Fisher). The medium was diluted with 50% fresh medium every 3 days.

### Brain stereotactic injection

The mice were anesthetized by intraperitoneal injection of 1.25% tribromoethanol at a dose of 0.2 ml/10 g and placed on a heating pad to maintain body temperature at 37 °C. Then, the mice were fixed to an operating table with ear bar adapters, and tweezers were used to fix the mouse tongue to prevent suffocation. After sterilizing with 75% alcohol, an incision was made in the mouse scalp, the skull was exposed, the bregma was located, a brain stereotaxic injection instrument was used to locate the proper coordinates for needle insertion (from bregma: AP: -2.2 ML: ±2.0 DV: -2.0), and AAV was injected into the hippocampal region of the mice [14]. AAV9-CMV-SaCas9-EGFP-U6-gRNA (purchased from Weizhen Biotechnology Company) (OV-scramble) and AAV9-CMV-Rtn4A-OV-U6-gRNA (purchased from Weizhen Biotechnology Company) (Rtn4A-AAV-OV) were injected into C57 mice. rAAV-U6-shRNA (scramble)-CMV-EGFP-pA (purchased from BrainVTA Biotechnology) was injected into the WT (KD-scramble) and Tau (KD-scramble) groups. rAAV-U6-shRNA3 (Rtn4A)-CMV-EGFP-pA (purchased from BrainVTA Biotechnology) was injected into the WT (Rtn4A-AAV-KD) and Tau (Rtn4A-AAV-KD) groups. A mouse skull drill was used to make a hole in the skull at the needle insertion site, a microinjection pump was used to inject 0.5 µl of adenovirus into the hippocampus of one hemisphere at a speed of 0.1 µl/min, and the needle was kept in place for 30 min after adenovirus injection. Then, AAV was injected into the hippocampus of the other hemisphere following the same method. After AAV was injected into the hippocampus of both hemispheres, the mice were removed from the brain stereotaxic injection instrument. The mouse scalp was sutured, and the mice were placed in a rat cage until they awoke.

### Western blotting

Before the experiment, a lysis buffer was prepared by mixing RIPA buffer (CST) with protease inhibitor cocktail (CST) and a phosphatase inhibitor cocktail (Sigma). Then, the tissue was lysed and homogenized. After 30 min, the supernatant of the tissue homogenate was collected after centrifugation at 12,000×g at 4 °C for 15 min, and the total protein concentration was determined using a spectrophotometer (Thermo Fisher). Equal amounts of protein samples were quantified by using the BCA protein quantification kit (Thermo Fisher), The supernatant was mixed with 4× loading buffer (Thermo Fisher) and boiled for 5 min at 100 °C to acquire samples for WB analysis. The samples were electrophoretically separated on SDS-PAGE gels (Bio-Rad) and then transferred to polyvinylidene difluoride membranes. Afterward, the membranes were blocked with 5% BSA for 1 h. Then, the membranes were washed with Tris-buffered saline containing Tween-20 (TBST, 0.1% Tween-20). Next, the membranes were incubated with one of the following primary antibodies against the following proteins overnight at 4 °C: Nogo-A (Abcam, 1:1000, Cat: #ab62024), Tau (CST, 1:1000, Cat: #46687), β-Actin (CST, 1:1000, Cat: #4967), phospho-tau (Thr181) (CST, 1:1000, Cat: #12885), phospho-tau (Thr205) (CST, 1:1000, Cat: #49561), phospho-tau (Ser404) (CST, 1:1000, Cat: #20194), phospho-tau (Thr231) (Abcam, 1:1000, Cat: #ab151559), phospho-tau (Ser262) (Thermo Fisher, 1:1000, Cat: #44-750G), phospho-tau (Thr396) (Sigma, 1:1000, Cat: #AB9658), phospho-tau (Ser202/ Thr205) (CST, 1:1000, Cat: #30505), phospho-AKT (Ser473) (CST, 1:1000, Cat: #9271), GAPDH (CST, 1:1000, Cat: #2118), ROCK2 (Sigma, 1:1000, Cat: #HPA007459), PSD95 (Abcam, 1:1000, Cat: # ab238135), AKT (CST, 1:1000, Cat: #9272), PI3K p85 (CST, 1:1000, Cat: #4292), phospho-GSK3β (Ser9) (CST, 1:1000, Cat:#5558), GSK3β (CST, 1:1000, Cat: #12456), Synaptophysin (Abcam, 1:1000, Cat:ab32127 ), The next day, the membranes were washed with Tris-buffered saline with Tween-20 (TBST, 0.1% Tween-20) followed by incubation with a secondary antibody (peroxidase conjugated-AffiniPure goat anti-rabbit/mouse, 1:5000, CST, Cat: #7074, #43593) for 1 h at room temperature. Finally, the membranes were washed with TBST and immersed in enhanced chemiluminescence (ECL) detection reagent (Millipore, Cat: Wbkls0100) before the signals were detected, and photos were captured using an imaging system.

### Open field test

The open field test was used to assess anxiety and depression in mice, as described previously [15]. The open field test consisted of a 40 cm×40 cm×40 cm×40 cm white box without a lid, and the EthoVision XT behavioral analysis system was used to record and analyze the data. A camera connected to the EthoVision XT behavioral analysis system was placed above the box to record the mouse trajectories and analyze the data. The environment was kept quiet during the test, and the room temperature was maintained at 23±1 °C. First, each mouse was permitted to acclimate to the box for 10 min, and EthoVision XT software was used to record its trajectory in the box. After each mouse finished a trial, the box was cleaned with 75% alcohol, and the smell of alcohol was allowed to dissipate. After all the mice were tested, EthoVision XT software was used to analyze the trajectories of the mice. The bottom of the box was divided into nine squares of uniform size, and the time spent in and number of entries into the central area were determined.

### Y maze test

This experiment was performed to evaluate whether Nogo-A influences short-term memory deficits in C57 and hTau mice. P301S mice were generated as described previously [16]. The three arms of the Y-maze were all 50 cm in length, and the angle between two adjacent arms was 120°. A camera directly connected to the EthoVision XT behavioral analysis system was placed directly above the Y-maze to record the trajectories of the mice and analyze the data. The environment was kept quiet during the test, and the room temperature was maintained at 23±1 °C. Each mouse was placed in the center of the Y-maze, and EthoVision XT software was used to record its trajectory in the Y-maze for 10 minutes. After the mouse finished the test, the Y-maze was cleaned with 75% alcohol, and the next mouse was placed in the maze after the alcohol smell dissipated. After all the mice completed the test, we used EthoVision XT software to analyze spontaneous alternation behavior and calculated the spontaneous alternation percentage (actual alternation/ maximum alternation x 100%) for each mouse.

### Novel object recognition (NOR) test and novel object location (NOL) test

This experiment was performed to evaluate whether Nogo-A deficiency influences the learning and memory of novel objects and novel locations in C57 and hTau mice. P301S mice, as described previously [17]. The NOR test was performed in a white plastic box (40 × 40 × 40 cm) illuminated by an overhead light (40 lux), and the room temperature (25 °C) was kept constant throughout the test. First, a mouse was permitted to acclimate to the box for 10 min, and then it was returned to its cage for 5 min of rest. Afterward, the training session began. Three identical objects (diameter of 4 cm and height of 2.8 cm) were placed in 3 corners of the arena 8 cm from the wall [18], and each mouse was permitted to explore them for 10 min. Twenty-four hours later, we moved one of the familiar objects to a new corner, and each mouse was permitted to explore the object for 10 min. Then, we replaced one of the familiar objects with a novel object without changing the locations of the objects. Each mouse was returned to the box and allowed to explore the objects for 10 min. The total distance traveled, and time spent sniffing each object were analyzed using TopScan 3.0 software (Clever Sys Inc.).

### Morris water maze (MWM)

The Morris water maze (MWM) was used to evaluate whether Nogo-A influences learning and memory deficits in C57 and hTau mice. P301S mice. The MWM test consisted of place navigation and spatial probe tests and was performed as previously described [19, 20], with appropriate modifications. The apparatus was composed of a circular pool (diameter: 120 cm) containing 30 cm of water (22 ± 1 °C) and a steel platform (diameter: 10 cm). The circular pool was divided into four equal quadrants, and the steel platform was positioned in the center of one of the quadrants. Throughout the experiment, the room temperature (25 °C), humidity (60-80%), and all the objects in the room were kept constant. The place navigation test lasted for 7 days, and every mouse underwent three training sessions in which they were released into a different quadrant every day (each training session was called a trial, and the intertrial interval was 1 min). From the first day to the seventh day (day 1-day 7), the platform was hidden 1.5 cm below the water surface. In each trial, the mice were placed in the pool and allowed to search for the platform. If the mouse found the platform within 60 s, the time needed to reach the platform was recorded as the escape latency by a behavioral analysis system (Shanghai Jiliang Technology). If the mouse did not find the platform within 60 s, it was gently led to the platform and allowed to remain on it for 15 s, and the escape time was recorded as 60 s. On the eighth day (day 8), the platform was removed, and the spatial probe test was conducted. The mice were forced to swim in the pool for 60 s, and the time spent in the target quadrant, the number of times the animal crossed the previous area of the platform, and the distance traveled in the target quadrant were recorded.

### Immunofluorescence

Briefly, the left hemisphere of the brain was fixed with 4% paraformaldehyde. Then, the samples were cut into frozen sections (with a thickness of 6~15 μm). The frozen sections were placed at 37 °C for half an hour, and then the brain sections were circled with an immunohistochemical pen and incubated for 10 minutes. Then, the sections were blocked with Super Blocking Buffer at room temperature for 1 h, and primary antibodies against GFP (1:100, Abcam, Cat:ab290), phospho-tau (Ser202/Thr205) (CST, 1:1000, Cat:# 30505), Meanwhile, we also established a control group in which only secondary antibodies were added, and PBST was used instead of primary antibodies for slide processing. Finally, the non-specific binding caused by secondary antibodies was removed based on the results. Diluted in Super Blocking Buffer were added and incubated at 4 °C in the dark for 48 h. The sections were washed three times with 0.3% PBST for 5 min each next day. Then, a corresponding fluorescent secondary antibody (Abcam, 1:1000, Cat: ab150077, ab150116) was added, and the sections were incubated at room temperature for 2 h in the dark. Finally, 150 µl of anti-fluorescence quenching agent (Southern Biotech) was added dropwise to the glass slides, and cover glasses were used to cover the slides. Images were obtained with an Axiovert 200 M microscope (Zeiss) or a confocal microscope (Leica).

### Golgi staining

Golgi staining of mouse brains was performed using the FD Rapid GolgiStainTM Kit from FD Neuro Technologies. First, the impregnation solution was prepared by mixing equal volumes of Solutions A and B for at least 24 hours prior to use and left unstirred. Then, the mice were sacrificed, and the brains were removed rapidly. Brain tissue samples were immersed in impregnation solution at room temperature for 2 weeks in the dark. The impregnation solution was replaced the next day. After 2 weeks, the brain tissue samples were transferred to Solution C and stored at room temperature in the dark for 72 hours. Solution C was replaced the next day. After 3 days, the soaked tissues were removed and sliced into 150 μm thick coronal sections using a vibrating microtome. Next, the sections were placed in staining solution, which consisted of 1 part Solution D, 1 part Solution E, and 2 parts double distilled for 10 min, followed by dehydration with 50%, 75%, 95%, and 100% ethanol, and finally transferred to xylene. The sections were covered with neutral resin and sealed with a coverslip. Images were acquired using a vertical fluorescence microscope, and the density of dendritic spines in the DG region was analyzed using ImageJ software.

**Figure 1.**
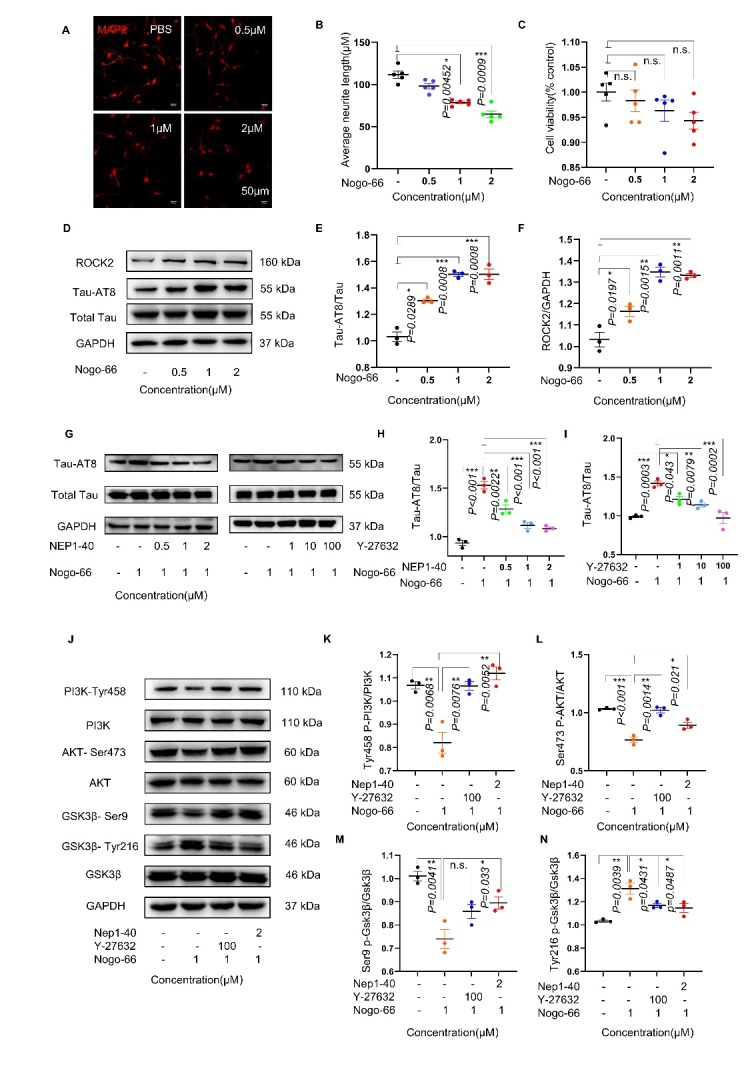
**Nogo-66 induced tau phosphorylation via the ROCK/AKT/GSK3β signaling pathway in cortical neurons of SD rats**. (A, B) Representative immunofluorescence image showing the effect of Nogo-66-mediated inhibition of neurite outgrowth in cortical neurons. n=5 in each group. (**C**) Cell viability of Nogo-66-treated neurons, as determined by CCK-8 assay; n=5 in each group. (D, E) Western blot analysis of tau phosphorylation at AT8 sites after treatment with different concentrations of Nogo-66; n=3 in each group. (**F**) ROCK2 protein expression levels were examined by Western blotting and quantified with ImageJ software (n= 3 in each group). (**G-I**) Western blotting was used to detect the effect of the Nogo-66 receptor antagonist NEP1-40 and the ROCK inhibitor Y-27632 on tau phosphorylation at the AT8 site; n=3 in each group. (**J**) Western blots showing the levels of different proteins in the PI3K/AKT/GSK3β signaling pathway in primary cultured cortical cells. (**K-N**) The data are presented as the average ± S.E.M. (n=3 in each group). Each point represents an independent experiment. Significance was conducted Kruskal-Wallis test, followed by Dunn’s multiple comparisons with a significant difference set at 0.05. n.s.=not significant, * *p* < 0.05, ** *p* < 0.01, *** *p* < 0.001.

### Statistical analysis

We performed a Shapiro-Wilk test to check for normal Gaussian distribution. If the data followed a normal distribution, we used Tukey's multiple comparisons tests for one-way ANOVA. If the data did not follow a normal distribution, we used the Kruskal-Wallis test, followed by Dunn's multiple comparisons. For smaller sample sizes (N<6), we conducted non-parametric tests using the Wilcoxon test. The MWM test data were analyzed using two-way repeated-measures ANOVA. T tests and one-way ANOVA were applied to compare two independent groups and three or more groups, respectively. The data are presented as the means ± standard errors of the means (SEMs), and differences were considered statistically significant when *P* < 0.05.

## RESULTS

### Nogo-66 increased tau phosphorylation via the ROCK/AKT/GSK3 β pathway in rat cortical neurons

To investigate the effect of Nogo-A on tau phosphorylation in neurons, the cortical neurons of neonatal SD rats were treated with the Nogo-A active fragment Nogo-66 at different concentrations (0.5, 1 and 2 μM) for 48 hours, and then, neuronal survival and morphology were examined by the CCK-8 assay and IF staining. Compared with PBS, Nogo-66 decreased the length of MAP2-positive dendrites in a dose-dependent manner (Fig. 1A and 1B), and the addition of Nogo-66 at the tested concentrations did not affect the neuronal survival rate (Fig. 1C). In addition, treatment with different concentrations of Nogo-66 differentially regulated the levels of P-tauAT8 compared with those in the control group, while 0.5 μM, 1 μM and 2 μM Nogo-66 significantly increased P-tauAT8 levels (Fig. 1D and 1E). In addition, 1 μM and 2 μM Nogo-66 increased the expression of ROCK2 (Fig. 1D and 1F).

To determine whether Nogo-A regulates tau phosphorylation via its receptor, we pretreated cortical neurons with NEP1-40, a Nogo-A receptor antagonist. We found that the effect of Nogo-66 on P-tauAT8 was abolished by treatment with NEP1-40 (Fig. 1G and 1H). Given that Nogo-66 treatment increased ROCK2 expression, we examined whether ROCK is involved in Nogo-induced tau phosphorylation and observed that Nogo-66 failed to increase P-tauAT8 levels in primary neurons pretreated with the ROCK inhibitor Y-27632 at different concentrations (1 μM, 10 μM and 100 μM) (Fig. 1g and 1i).

We further explored the changes in the levels of proteins related to possible signaling pathways involved in Nogo-A-regulated tau phosphorylation. The Western blot results showed that treatment of primary neurons with Nogo-66 decreased the levels of PI3K phosphorylated at Tyr458 (P-PI3K Y458), AKT phosphorylated at Ser473 (P-AKT S473) and GSK3β phosphorylated at Ser9 (P-GSK3β S9) but increased the levels of GSK3β phosphorylated at Tyr216 (P-GSK3β Y216) compared to those in the control group (Fig. 1G). Interestingly, pretreatment with either NEP1-40 or Y-27632 eliminated these effects induced by Nogo-66. (Fig. 1J-N).

### Nogo-A overexpression impaired the learning and memory abilities of C57BL/6 N mice

**Figure 2.**
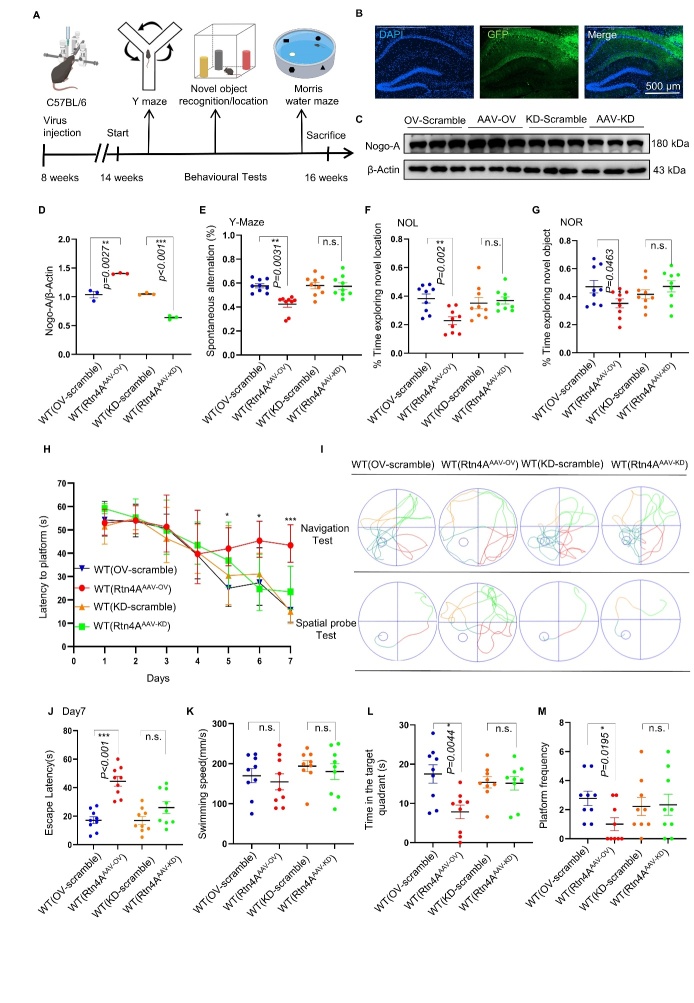
**Overexpression of Nogo-A aggravated cognitive deficits in C57BL/6 N mice**. (**A**) Schematic illustration of the workflow and data analysis. (**B**) Confocal image of an AAV-injected hippocampus labeled with GFP (green) (40x). (C, D) Representative western blots showing the levels of Nogo-A proteins in the hippocampus of WT (scramble), WT (Rtn4A-AAV-OV), and WT (Rtn4A-AAV-KD) mice; n=3 for each group. (**E**) Number of spontaneous alternations in the Y-maze test; n=9 for each group. (F, G) Recognition indices of the NOL (F) and NOR (G) tests; n=9 for each group. (**H**) Latency to escape to a hidden platform in the MWM test during a 7-d training period; Day5: WT (scramble) versus WT(Rtn4A-AAV-OV), *P*=0.0123. Day6: WT (scramble) versus WT(Rtn4A-AAV-OV), *P*=0.0275. Day7: WT (scramble) versus WT(Rtn4A-AAV-OV), *P*< 0.001; (n = 9 mice in each group; *P< 0.05, **P< 0.01, ***P< 0.001; two-way ANOVA followed by Tukey’s multiple-comparisons test). (**I**) Swimming trajectories in the navigation test and spatial probe test. (**J**) The escape latency of each group was statistically analyzed on day 7; n=9 for each group. (**K**) The average speed of the mice in the spatial probe test. (**L**) Time spent in the target quadrant in the probe trial; n=9 for each group. (**M**) The number of times the mice passed through the platform location in the probe trial, n=9 for each group. Each point represents an individual animal. Data sets were tested for normal Gaussian distribution via Shapiro-Wilk test. Significance was determined by ANOVA, Tukey's multiple comparisons or Kruskal-Wallis test, followed by Dunn’s multiple comparisons with a significant difference set at 0.05. n.s.=not significant, * *p* < 0.05, ** *p* < 0.01, *** *p* < 0.001.

To determine whether Nogo-A affects the cognitive function of C57 mice, we modulated Nogo-A expression in the hippocampus of C57 mice via AAV. Four different types of AAVs were injected into the hippocampus of C57 mice at 8 weeks of age (Fig. 2A). Confocal imaging revealed that the viruses were expressed in the hippocampus at 6 weeks postinjection (Fig. 2B). Immunoblot analyses revealed that WT (Rtn4A-AAV-OV) upregulated whereas WT (Rtn4A-AAV-KD) downregulated the expression of Nogo-A (Fig. 2C and 2D).

Next, behavioral tests were performed to assess the cognitive function of the experimental mice. Neither WT (Rtn4A-AAV-OV) nor WT (Rtn4A-AAV-KD) injection significantly affected the performance of WT C57BL/6J mice in the open field (OF) test (Supplementary Fig. 1A-D), suggesting that Nogo-A has no effect on anxiety in WT mice. In the Y maze test, the spontaneous alternation score was reduced in the WT (Rtn4A-AAV-OV) group but not in the WT (Rtn4A-AAV-KD) group (Fig. 2E). These data indicate that upregulation of Nogo-A impairs short-term spatial learning and memory in mice.

In the NOR/NOL test, mice in the WT (Rtn4A-AAV-OV) group spent less time exploring the object in the novel location than did those in the control group in the NOL test (Fig. 2F). Similarly, the WT (Rtn4A-AAV-OV) group spent less time exploring the novel object than did the control group (Fig. 2G), whereas there was no significant difference in the amount of time spent exploring the new object between the WT (Rtn4A-AAV-KD) group and the control group (Fig. 2G). This finding suggested that overexpression of Nogo-A results in recognition memory deficits in mice.

Next, we performed the Morris water maze (MWM) test to assess the cognitive ability of the experimental mice. The experimental diagram is shown in Supplementary Fig. 1E. During the training phase, the escape latency significantly decreased from day 1 to day 7 in all groups except for the WT (Rtn4A-AAV-OV) group (Figs. 2H-J and Supplementary Fig. 1F-G). In the spatial probe test, there was no significant difference in swimming speed among the groups (Fig. 2K). Mice in the WT (Rtn4A-AAV-OV) group spent significantly less time in the target quadrant than did those in the WT (OV-scramble) group (Fig. 2L). In addition, fewer mice in the WT (Rtn4A-AAV-OV) group crossed the area where the platform was previously located than did those in the WT (OV-scramble) group (Fig. 2M). These results demonstrate that overexpression of Nogo-A impairs spatial learning and memory in mice.

### Overexpression of Nogo-A increased tau phosphorylation, reduced dendritic spine density and increased synapse deficits in the hippocampus of C57BL/6J mice

**Figure 3.**
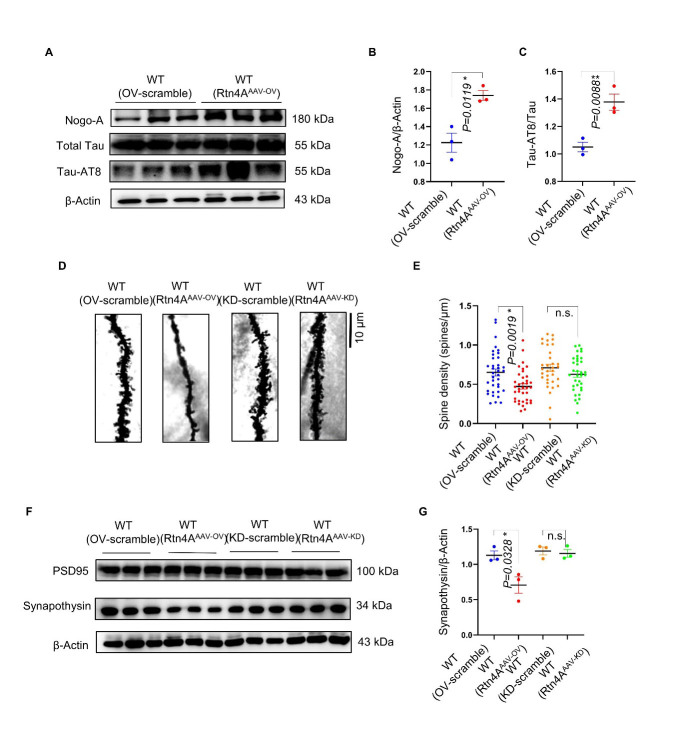
**Overexpression of Nogo-A increased tau phosphorylation at the AT8 site, decreased dendrite density and aggravated synaptic loss in C57BL/6 N mice**. (**A**) Representative western blots showing the levels of Nogo-A and tau phosphorylation at AT8. (B, C) Statistical analysis of Nogo-A and tau phosphorylation at AT8 sites; n=3 for each group. (D, E) Representative images and quantification of dendritic spine density. Scale bars = 10 μm, n= 3 mice per group. (**F**) Representative western blots showing the levels of synaptophysin and PSD95 in the hippocampus. (**G**) The expression levels of synaptophysin in the hippocampus were examined by Western blotting and quantified with ImageJ software (n= 3 mice per group). Each point represents an individual animal. Normality was tested for using Shapiro-Wilk test, and significance was tested using Kruskal- Wallis test, followed by Dunn’s multiple comparisons as these data set were not gaussian. The data are presented as the means ± S.E.Ms. n.s.=not significant, * *p* < 0.05, ** *p* < 0.01, *** *p* < 0.001.

The levels of tau phosphorylated at AT8 (P-tauAT8) in the mouse hippocampus were determined by Western blotting (Fig. 3A). The results showed that the levels of P-tauAT8 were significantly greater in the hippocampus of mice in the WT (Rtn4A-AAV-OV) group than in those in the WT (OV-scramble) group (Fig. 3B and 3C). There was no significant difference in hippocampal P-tauAT8 levels between the WT (Rtn4A-AAV-KD) and WT (KD-scramble) groups (Fig. S1H). Therefore, the observed reductions in learning and memory abilities may have been due to increased levels of tau phosphorylation. In addition, we observed that the density of dendritic spines on hippocampal neurons in the WT (Rtn4A-AAV-OV) group was significantly lower than that in the WT (OV-scramble) group (Fig. 3D and 3E). There was no significant difference in the density of dendritic spines between the WT (Rtn4A-AAV-KD) and WT (KD-scramble) groups (Fig. 3E). In addition, Western blot analysis showed that synaptophysin expression dramatically decreased in the hippocampus of the mice in the WT (Rtn4A-AAV-OV) group (Fig. 3F and 3G). These results suggest that tau phosphorylation and synaptic deficits may contribute to the cognitive impairment induced by Nogo-A overexpression.

### Knockdown of Nogo-A attenuated the learning and memory impairments induced by hTau. P301S mice

**Figure 4.**
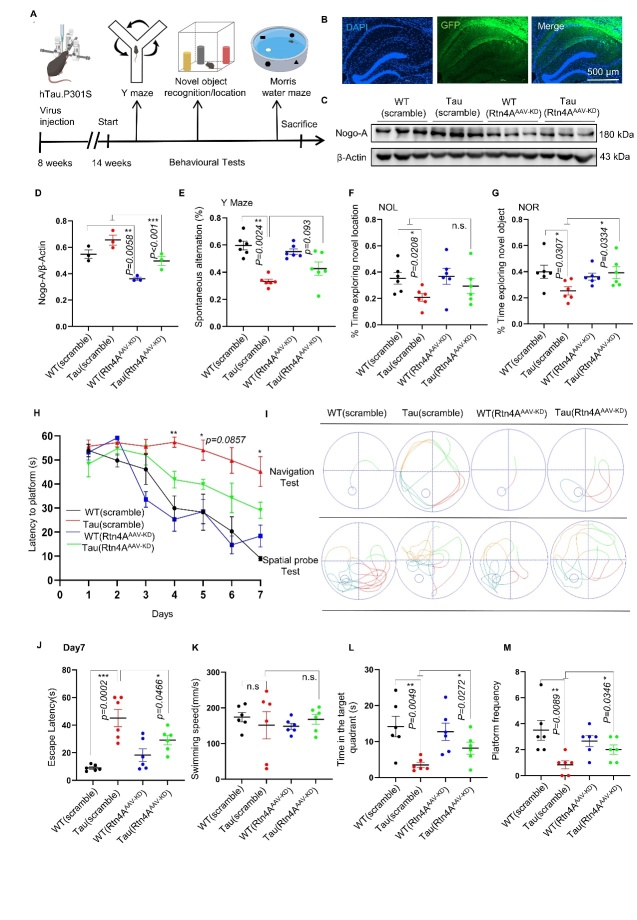
**Knockdown of Nogo-A ameliorated the cognitive decline induced by hTau**. P301S mice. (**A**) Schematic diagram of the experimental process. (**B**) Confocal image of an AAV-injected hippocampus labeled with GFP (green) (40x). (C, D) Representative western blots showing the levels of Nogo-A proteins in the hippocampus of WT (scramble), Tau (scramble), WT (Rtn4A-AAV-KD), and Tau (Rtn4A-AAV-KD) mice; n=3 for each group. (**E**) The spontaneous alternation of each group was statistically analyzed. n=6 for each group. (**F**) Discrimination ratio of mice in the novel location recognition (NOL) test; n=6 for each group. (**G**) Discrimination ratio of mice in the NOR test; n=6 for each group. (**H**) Latency to escape to a hidden platform in the MWM test during a 7-d training period; Day4: Tau(scramble) versus Tau (Rtn4A-AAV-KD), *P*=0.0033; Day5: Tau(scramble) versus Tau (Rtn4A-AAV-KD), *P*=0.0129; Day6: Tau(scramble) versus Tau (Rtn4A-AAV-KD), *P*=0.0857. Day7: Tau(scramble) versus Tau (Rtn4A-AAV-KD), *P*=0.0466; (n=6 mice in each group; *P< 0.05, **P< 0.01, ***P< 0.001; two-way ANOVA followed by Tukey’s multiple-comparisons test). (**I**) Representative swimming trajectories in the navigation test and spatial probe test. (**J**) The escape latency of each group was statistically analyzed on day 7; n=6 for each group. (**K**) The average speed of the mice in the spatial probe test. n=6 for each group. (**L**) Percentage of time spent in the target quadrant. n=6 for each group. (**M**) The number of times the mice crossed the location of the removed platform. n=6 for each group). Each point represents an individual animal. Data sets were tested for normal Gaussian distribution via Shapiro-Wilk test. Significance was determined by ANOVA, Tukey's multiple comparisons or Kruskal-Wallis test, followed by Dunn’s multiple comparisons with a significant difference set at 0.05. n.s.=not significant, * *p* < 0.05, ** *p* < 0.01, *** *p* < 0.001.

To investigate whether knockdown of Nogo-A could alleviate learning and memory impairment in hTau. P301S transgenic mice, the transgenic mice were injected with a Nogo-A knockdown AAV at 9 months of age (Fig. 4A). After 45 days of infection, the expression of the virus was confirmed by confocal imaging (Fig. 4B). The downregulation of Nogo-A in Rtn4A-AAV-KD-injected mice was verified by Western blotting (Figs. 4C and 4D). We then carried out behavioral tests to evaluate the brain function of the experimental mice.

In the OF test, there was no significant difference in time spent in the central area, number of entries into the central area, or average velocity traveled between the Tau (Rtn4A-AAV-KD) and Tau (KD-scramble) groups (Supplementary Fig. 2A-C). In the Y maze test, the spontaneous alternation score was improved to some extent in the Tau (Rtn4A-AAV-KD) group compared with the Tau (KD-scramble) group, although the difference did not reach statistical significance (Fig. 4E). After moving one of the original objects to a new location, there was no significant difference in the time spent exploring the object in the novel location between the tau (KD-scramble) group and the tau (Rtn4A-AAV-KD) group (Fig. 4F). In the NOR test, mice in the Tau (Rtn4A-AAV-KD) group spent more time exploring the novel object than did those in the Tau (KD-scramble) group (Fig. 4G). In the MWM test, the escape latency significantly decreased from day 1 to day 7 in all groups except the Tau (KD-scramble) group during the training phase (Fig. 4H-4J). In the spatial probe test, although there was no significant difference in swimming speed among the groups (Fig. 4K), mice in the Tau (Rtn4A-AAV-KD) group spent more time in the target quadrant than did those in the Tau (KD-scramble) group (Fig. 4I). Furthermore, mice in the Tau (Rtn4A-AAV-KD) group crossed the platform area more than did those in the Tau (KD-scramble) group (Fig. 4M). Taken together, these data indicate that knockdown of Nogo-A can improve the cognitive function of hTau. P301S mice.

### Knockdown of Nogo-A reduced tau phosphorylation, attenuated tau pathology, improved dendritic spine density and rescued synaptic deficits in hTau. P301S mice

**Figure 5.**
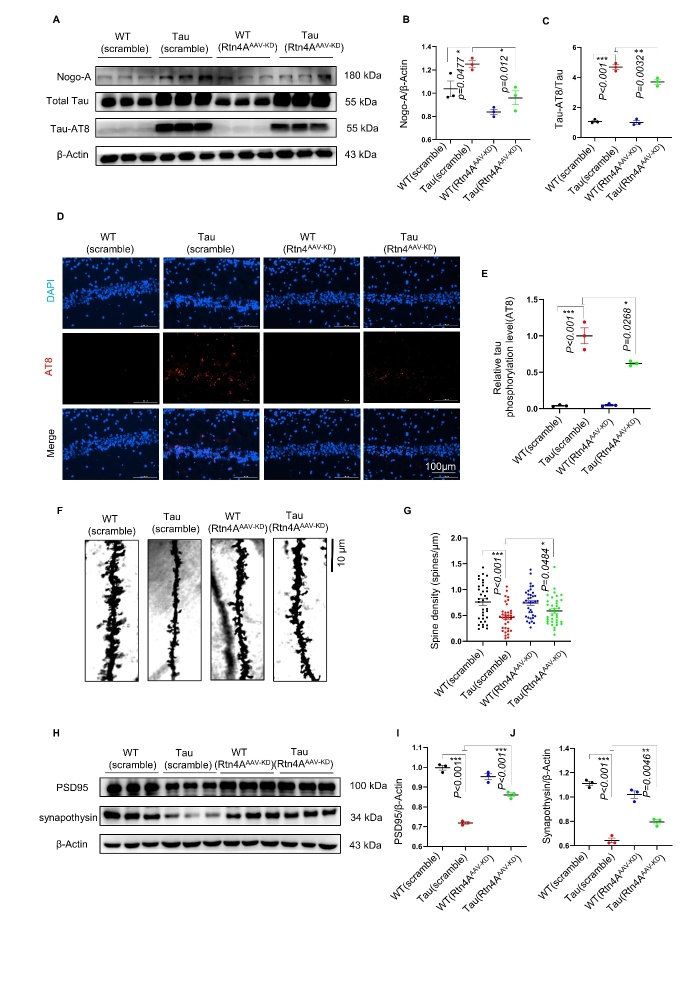
**Knockdown of Nogo-A reduced tau phosphorylation at AT8, tau pathology, dendrite density and synaptic loss in hTau**. P301S mice. (**A-C**) Representative western blots showing Nogo-A and tau phosphorylation at AT8. n=3 for each group. (**D-E**) Representative immunofluorescence staining of AT8-positive cells in the hippocampus (100x); n=3 for each group. (F, G) Representative images and quantification of dendritic spine density. Scale bars=10 μm, n=3 mice per group. (**H-J**) Western blots showing the levels of synaptophysin and PSD95 in hippocampal tissue; n=3 for each group. Western blots were quantified by ImageJ software (n=3 mice per group). Each point represents an individual animal. Data sets were tested for normal Gaussian distribution via Shapiro-Wilk test. Significance was determined by Kruskal-Wallis test, followed by Dunn’s multiple comparisons with a significant difference set at 0.05. n.s.=not significant, * *p* < 0.05, ** *p* < 0.01, *** *p* < 0.001.

Western blotting was performed, and the results showed that the levels of Nogo-A and P-tauAT8 dramatically decreased in the hippocampi of mice in the Tau (Rtn4A-AAV-KD) group (Fig. 5A-5C). Immunofluorescence (IF) staining confirmed the reduction in AT8+ levels in the hippocampus of mice in the Tau (Rtn4A-AAV-KD) group (Fig. 5D and 5E). To visualize neuronal dendrites, we carried out Golgi staining and observed that the density of dendritic spines in the hippocampi of mice in the Tau (Rtn4A-AAV-KD) group was significantly greater than that in the hippocampi of mice in the Tau (KD-scramble) group (Figs. 5F and 5G). Furthermore, Western blot analysis revealed that the levels of synaptophysin (Figs. 5H and 5I) and PSD95 (Figs. 5H and 5J) were significantly greater in hippocampal samples from the Tau (Rtn4A-AAV-KD) group than in those from the Tau (KD-scramble) group.

### The ROCK/AKT/GSKβ pathway was activated in Nogo-A-mediated regulation of tau phosphorylation in vivo

**Figure 6.**
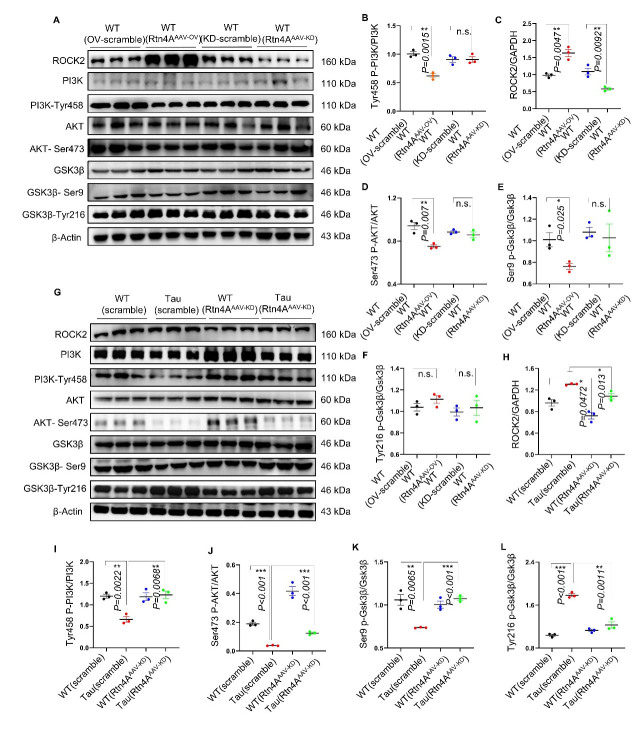
**Nogo-A promoted tauopathy vulnerability via the ROCK/AKT/GSK3β pathway in vivo**. (**A**) Western blots showing the levels of different proteins in the PI3K/AKT/GSK3β signaling pathway in C57BL/6 N mice. (**B-F**) The Data sets were tested for normal Gaussian distribution via Shapiro-Wilk test. Significance was determined by Kruskal-Wallis test, followed by Dunn’s multiple comparisons with a significant difference set at 0.05. n.s.=not significant, * *p* < 0.05, ** *p* < 0.01, *** *p* < 0.001. (**G**) Western blots showing the levels of different proteins in the PI3K/AKT/GSK3β signaling pathway in hTau. P301S mice. (**H-L**) Data sets were tested for normal Gaussian distribution via Shapiro-Wilk test. Significance was determined by Kruskal-Wallis test, followed by Dunn’s multiple comparisons with a significant difference set at 0.05. n.s.=not significant, * *p* < 0.05, ** *p* < 0.01, *** *p* < 0.001. n=3 for each group. Each point represents an individual animal.

We finally validated the signaling pathway involved in the effects of Nogo-A in cells using mouse hippocampal tissue. The Western blot results showed that overexpression of RTN4A decreased the levels of P-PI3K Y458, P-AKT S473 and P-GSK3β S9 but increased ROCK2 expression in hippocampal samples from C57 mice (Fig. 6A-F). In addition, we found that the levels of ROCK2 and P-GSK3β Y216 were increased, whereas the levels of P-AKTS473 and P-GSK3βS9 were decreased in the hippocampus of hTau. P301S mice compared with those of WT control mice. Strikingly, AAV-mediated downregulation of RTN4A reversed these alterations in the brains of the Tau-Tg mice (Fig. 6G-L). Taken together, the above data indicate that Nogo-A activates the Nogo-A/ROCK2/PI3K/AKT/GSK3β pathway, increases tau phosphorylation and leads to the degeneration of dendrites in the hippocampus in hTau. P301S mice.

**Table 1:** Comparison of Tau phosphorylation sites under 3 different models.

Tau phosphorylation sites	Cortical neurons of SD rats treated with Nogo-66	Wildtype mouse with overexpression of Nogo-A	hTau.P301S mouse with Knockdown of Nogo-A
**AT8**	++	++	++
**Ser202**	++	+	++
**Thr181**	-	+	+
**Ser262**	+	-	-
**Thr231**	NA	-	++
**Ser404**	-	-	++
**Ser199**	NA	-	-
**Ser396**	-	-	-

Notes: "-" means negative change; “+” means positive change; “++” means more serious positive change; “NA” means uncertainty

## DISCUSSION

The expression of the myelin protein Nogo-A has been reported to be increased in the brains of AD patients [10-12], suggesting that Nogo-A plays a role in the pathophysiology of Alzheimer's disease (AD). NFTs composed of the hyperphosphorylated form of tau are a classic feature of a class of disorders referred to as tauopathies, such as AD, which is characterized by cognitive decline [21, 22]. We speculated that Nogo-A may be related to tauopathies. The present study explored the effect of Nogo-A on tau hyperphosphorylation and the underlying regulatory pathway involved.

First, we found that the addition of Nogo-66 increased the phosphorylation of tau at AT8 and Ser262 in rat primary cortical neurons, and we further verified the same phenomena in C57 mice (Table 1; Supplementary Fig. 3A and 3B). Nogo-A was overexpressed in the hippocampus of mice by stereotactic injection of adeno-associated virus (AAV) vectors. After injecting Nogo-A-overexpressing virus into the hippocampal region of C57BL/6J mice, the phosphorylation of tau at AT8 and Thr181 increased, but the increase in tau phosphorylation at Thr181 was not as obvious as that at AT8 (Table 1; Supplementary Figs. 3C and 3D). There was no significant increase in the phosphorylation of tau at Ser262, Ser199, Ser396, or other sites. The anti-phospho-tau Ser202/Thr205 (AT8) antibody detects the main form of pathological Tau, which includes tau phosphorylated at Ser202, Thr205, and Ser208, as well as pre-NFT aggregates in tissues. Tau phosphorylation at different sites is clearly involved in the early and late stages of NFT progression. Steen *et al*. reported excessive tau phosphorylation at 43-55 different sites in the brains of AD patients. The anti-phospho-tau Ser202/Thr205 (AT8) antibody detects tau phosphorylated at the most common phosphorylation site and is commonly used to label NFTs to assess the severity of AD [25]. However, tau phosphorylation at Ser262 does not decrease with the addition of inhibitors to cortical neurons, suggesting that differences in tau phosphorylation at Ser262 are not mediated by the Nogo-A receptor (NgR)/ROCK pathway. The aggregation of phosphorylated tau protein in neurons results in the formation of NFTs, which leads to neuronal dysfunction [26]. In addition to inducing tau phosphorylation, the upregulation of Nogo-A in the hippocampus of C57 mice exacerbates tau pathology and synapse loss and decreases dendritic spine density, which results in impaired cognitive function in C57 mice.

Since Nogo-A was proven to induce tau hyperphosphorylation and exacerbate AD-associated pathology and cognitive decline, downregulation of Nogo-A may reverse AD progression and be an effective strategy to treat AD. Therefore, the human tau-expressing transgenic mouse line hTau. P301S was used as an AD model in this study. These mice carry a mutated form of the human Tau protein called P301S, which leads to abnormal aggregation of hyperphosphorylated Tau in the nervous system. In addition, these mice exhibit neurodegenerative changes, such as decreased dendritic spine density and synaptic loss, during the aging process, simulating the pathological features of AD. Our study revealed that knockdown of Nogo-A can reduce tau phosphorylation in the hippocampus and tau pathology and improve learning and memory in hTau. P301S transgenic mice. In addition to a decrease in tau phosphorylation at AT8 and Thr181, we also found that tau phosphorylation at Thr231 and Ser404 was significantly reduced after the injection of Nogo-A knockdown virus into hTau. P301S mice (Table 1; Supplementary Fig. 4A-H). Consistent with previous experiments, the change in phosphorylation at AT8 was the most pronounced. In addition, the immune-fluorescence results revealed that knockdown of Nogo-A significantly reduced tau phosphorylation at AT8 and Thr231 in the hippocampal region in hTau. P301S mice, thereby ameliorating tau pathology. Previous research has confirmed that excessive phosphorylation at AT8, Thr181, Thr231, and Ser404 is considered indicative of the transition from mild cognitive impairment to Alzheimer's disease, indicating the progression of dementia [27, 28]. Tau is phosphorylated at AT8 both in vitro and in vivo, which suggests that AT8 may be a key site whose phosphorylation is regulated by Nogo-A. Hyperphosphorylated tau also interacts with Aβ, leading to neuronal loss and synaptic damage, ultimately resulting in a decrease in cognitive ability and quality of life in AD patients [23, 24]. In the present study, in addition to reducing tau phosphorylation, downregulation of Nogo-A in the hippocampus of C57BL/6J mice also decreased tau pathology, rescued synapse loss and dendritic spine loss, and attenuated cognitive dysfunction in aged transgenic tau mice. The results suggested that in hTau. In P301S transgenic mice, inhibiting excessive tau phosphorylation by downregulating Nogo-A is an effective strategy for the treatment of AD.

Next, we explored the mechanism underlying the effect of Nogo-A on tau phosphorylation. Nogo-A interacts with NgR to inhibit axonal sprouting and synaptic remodeling via the downstream Rho-ROCK pathway [29]. ROCK expression was confirmed to be increased in mice overexpressing Nogo-A. Consistent with the in vivo experiments, the addition of Nogo-66 significantly increased ROCK expression in rat cortical neurons, and this effect was abolished by the NgR antagonist NEP1-40. In contrast, Nogo-A knockdown in hTau. ROCK expression decreased in P301 s mice. Analysis of postmortem brain samples from AD patients revealed decreased phosphorylation levels of AKT [30]. A study of mice with conditional knockout of the three isoforms of AKT showed tau hyperphosphorylation, including Thr205, Thr231 and Ser396, in the brain [31]. The phosphorylation of PI3K at Tyr458 and that of AKT at Ser473 were increased in Nogo-A-overexpressing C57BL/6J mice. The phosphorylation of PI3K at Tyr458 and that of AKT at Ser473 were decreased in the hippocampal region of hTau. In P301S mice, this decrease in 19 phosphorylation was reversed by Nogo-A knockdown. Furthermore, we also found increased phosphorylation of PI3K at Tyr458 and increased phosphorylation of AKT at Ser473 after the addition of Nogo-66 to rat cortical neurons, a phenomenon that could also be reversed by the addition of the NgR antagonist NEP1-40 or the ROCK inhibitor Y-27632. GSK3β is a kinase known to regulate tau hyperphosphorylation [32, 33]. Therefore, we examined whether GSK3β is involved in Nogo-A-induced tau hyperphosphorylation. The results showed that the changes in GSK3β phosphorylation at Ser9 and Tyr216 were not as significant in WT mice (cortical neurons of SD rats and C57 mice) as in hTau mice. P301S mice. However, we still observed a decrease in the phosphorylation of GSK3β at Ser9 in the hippocampal region of C57BL/6J mice injected with the Nogo-A-overexpressing virus. Additionally, we also observed a decrease in the phosphorylation of GSK3β in the cortical neurons of rats after Nogo-66 treatment, and these changes were reversed by the addition of the ROCK inhibitor Y-27632. In AD (hTau. P301S mice), tau phosphorylation at Ser9 in the hippocampal region was increased after Nogo-A knockout, while phosphorylation of GSK3β at Tyr216 was decreased. The phosphorylation of Tyr216 can activate the kinase activity of GSK3β, thereby promoting the phosphorylation of the Tau protein [34]. The above results suggest that Nogo-A-induced tau phosphorylation may be mediated by the NgR/ROCK/ PI3K/AKT/GSK3β signaling pathway.

Taken together, the results of this study suggest that overexpression of Nogo-A increases tau phosphorylation, exacerbates tau pathology in the hippocampus of wild-type mice and causes cognitive impairment. On the other hand, knockdown of Nogo-A reduces tau phosphorylation in the hippocampal region and improves learning and memory in the hTau. P301 s transgenic mice. In vitro, the addition of Nogo-66 caused an increase in tau phosphorylation, and this effect was reversed when the corresponding blockers were added. Therefore, our data support a critical role for Nogo-A in driving Alzheimer’s disease progression by inducing Tau phosphorylation through the NgR/ROCK/PI3K/AKT/GSK3 β pathway. In addition, we discovered an interesting phenomenon in which Nogo-A was overexpressed in C57BL/6 mice and Nogo-A was knocked down in hTau mice. P301S mice exhibit different changes in tau phosphorylation, at AT8. It is currently unknown whether changes in the phosphorylation of these sites other than AT8 occur in conjunction with or are independent of changes in the phosphorylation of AT8, which needs to be further explored in future work. This study provides new insight into the potential of Nogo-A as a target for preventing and treating tauopathies and the related mechanisms.

## Supplementary material

The Supplementary data can be found online at: www.aginganddisease.org/EN/10.14336/AD.2024.0053.


