# Supplementary material for: Nogo-A Drives Alzheimer’s Disease Progression by Inducing Tauopathy Vulnerability
Source: Aging Dis. 2024 Apr 23;16(2):1199–215. doi: 10.14336/AD.2024.0053 (PMC11964429; doi:10.14336/AD.2024.0053)
Supplement: Supplementary file 1 [file AD-16-2-1199-s.pdf]

SUPPLEMENTARY DATA

Nogo-A Drives Alzheimer’s Disease Progression by Inducing Tauopathy Vulnerability

Zijian Wang, Jun-ping Pan, Jiayuan Geng, Shijie Lv, Guisi Chen, Nian Fang, Zheng Zhang, Junliang Li, Xinke Xu, Rui Wang, Qing Zheng, Li Yan, Guobing Chen, Fei Xiao

© 2024. Wang Z et al. Published online at http://www.aginganddisease.org/EN/10.14336/AD.2024.0053

SUPPLEMENTARY DATA

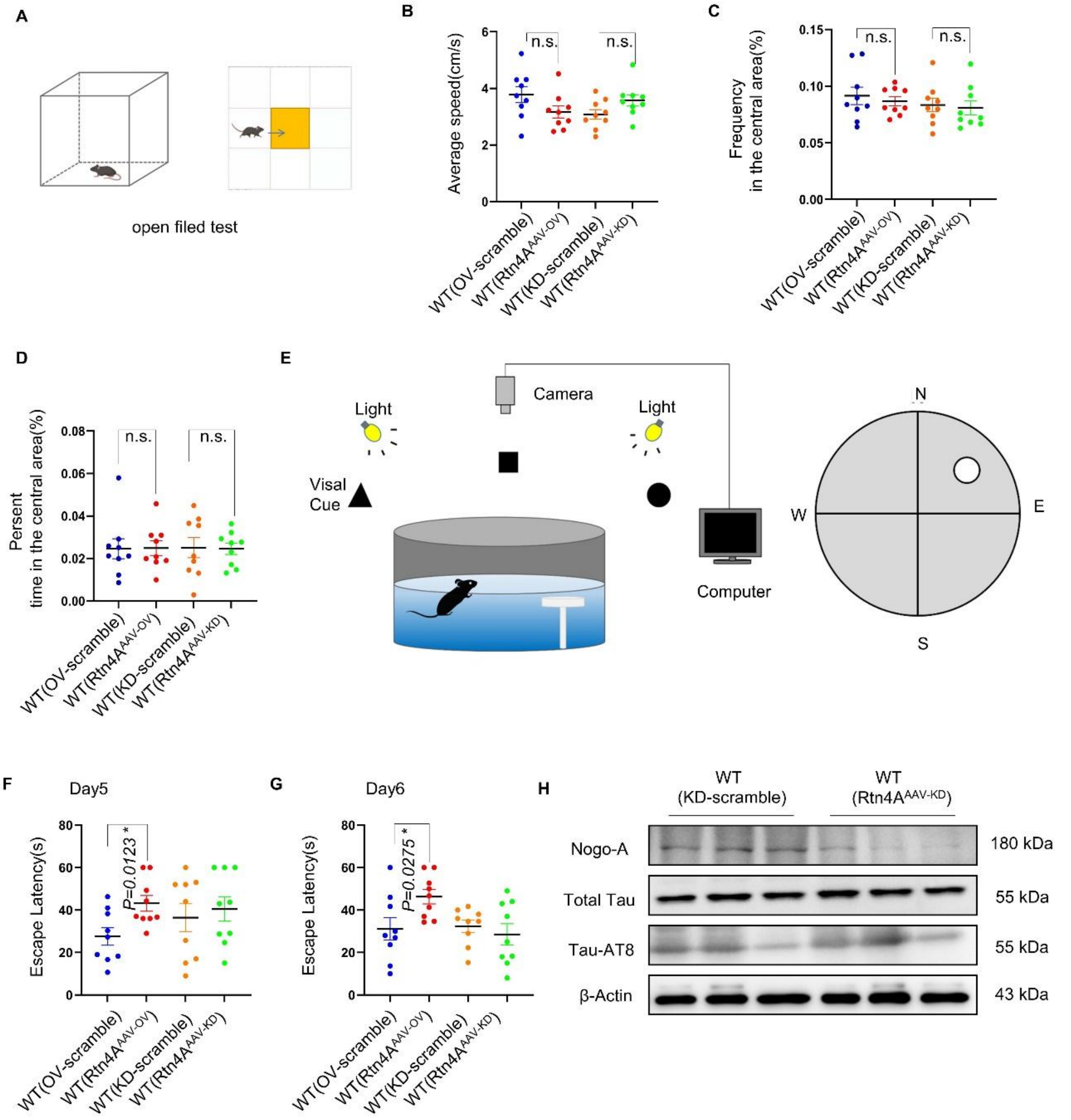

Supplementary Figure 1. Behavior and tau phosphorylation effects of Nogo-A overexpression or knockdown in C57BL/6 N mice. (A) Schematic diagram of the open field test. (**B**) The average speed of the mice, n=9 for each group. (**C-D**) The frequency (**C**) and time spent (**D**) in the central area of each group were statistically analyzed. n=3 for each group. (**E**) Schematic diagram of the Morris water maze. (**F-G**) The escape latency of each group was statistically analyzed on days 5 and 6. n=9 for each group. (**H**) Representative western blots showing the levels of Nogo-A and tau phosphorylation at AT8 sites in the WT (KD-scramble) group and WT (Rtn4AAAV-KD) group. Data sets were tested for normal Gaussian distribution via Shapiro-Wilk test. Significance was determined by Kruskal-Wallis test, followed by Dunn's multiple comparisons with a significant difference set at 0.05. n.s.=not significant, * p < 0.05, ** p < 0.01, *** p < 0.001. Each point represents an individual animal.

© 2024. Wang Z et al. Published online at http://www.aginganddisease.org/EN/10.14336/AD.2024.0053

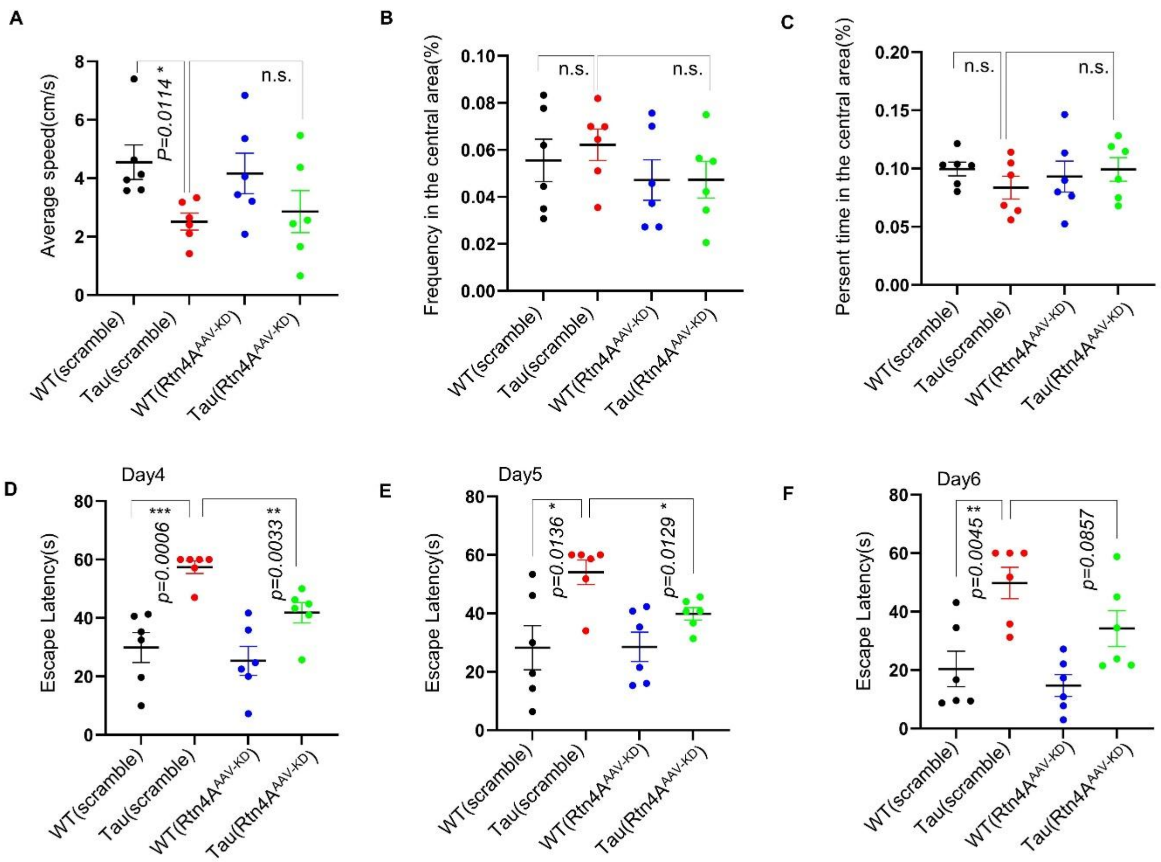

Supplementary Figure 2. Behavioral effects of Nogo-A knockdown in hTau. P301S mice. (A) The average speed of each group. n=6 mice per group. (**B-C**) The frequency (**B**) and the time spent (**C**) in the central area of each group were statistically analyzed. n=6 for each group. (**D**) The escape latency of each group was statistically analyzed on days 4, 5 and 6. *$p < 0.05$, **$p < 0.01$, and ***$p < 0.001$, groups analyzed by one-way ANOVA. Dunnett's multiple comparisons test followed by Tukey's multiple comparisons test were used for statistical analysis, with a significant difference set at 0.05. n.s.=not significant, * $p < 0.05$, ** $p < 0.01$, *** $p < 0.001$.

© 2024. Wang Z et al. Published online at http://www.aginganddisease.org/EN/10.14336/AD.2024.0053

SUPPLEMENTARY DATA

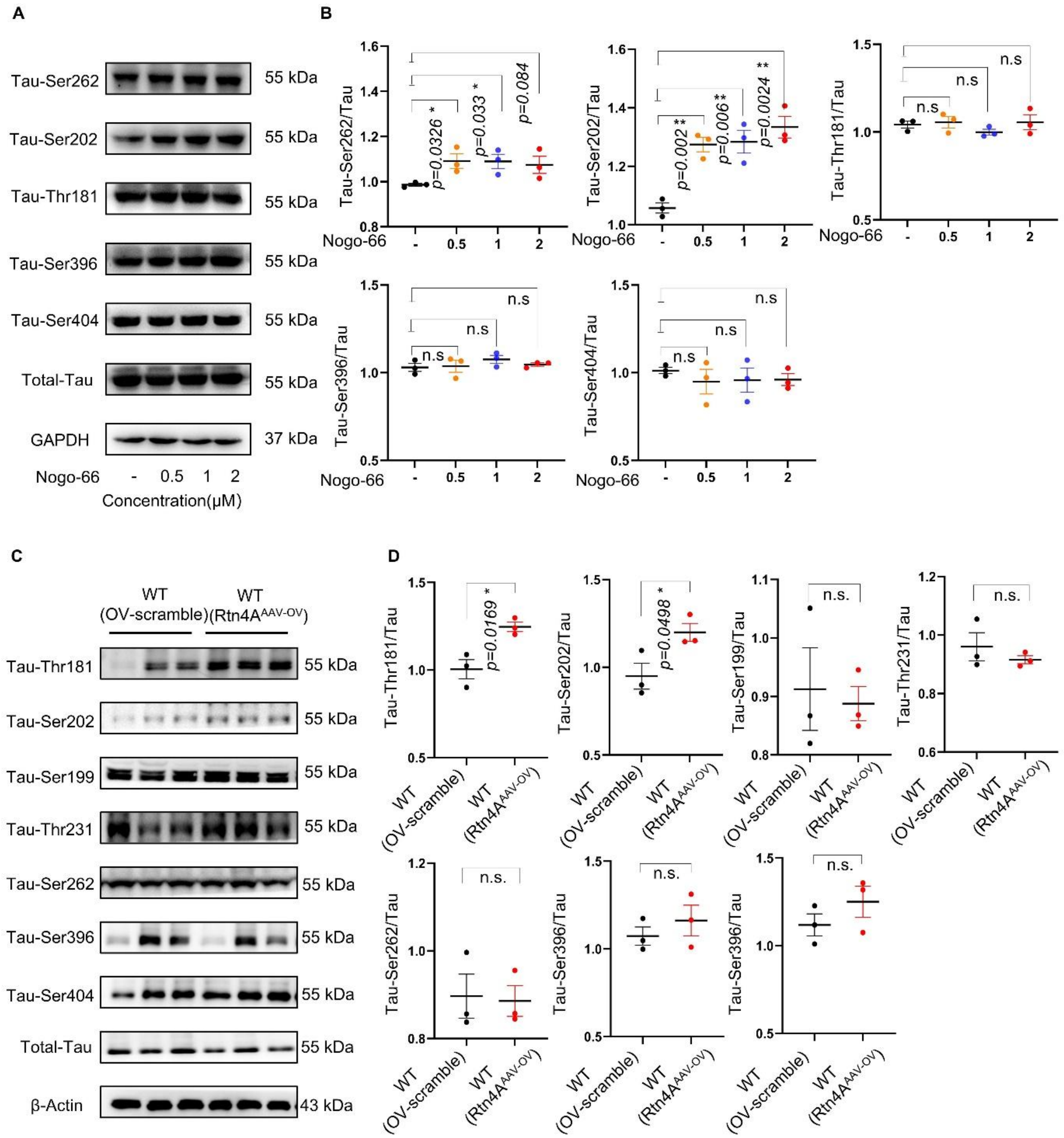

Supplementary Figure 3. Nogo-A overexpression promoted tau phosphorylation at multiple sites in rat cortical neurons and C57BL/6 N mice. (**A-B**) Western blots showing the levels of tau phosphorylation at Ser262, Ser202, Thr181, Ser396 and Ser404 at different concentrations of Nogo-66 in rat cortical neurons. n=3 in each group. (**C-D**) Western blots showing the levels of tau phosphorylation at Thr181, Ser202, Ser199, Thr231, Ser262, Ser396 and Ser404 in the hippocampus of the WT (scramble) and WT (Rtn4A-AAV-OV) groups. n=3 in each group. *$p < 0.05$, **$p < 0.01$, and ***$p < 0.001$, Data sets were tested for normal Gaussian distribution via Shapiro-Wilk test. Significance was determined by Kruskal-Wallis test, followed by Dunn's multiple comparisons with a significant difference set at 0.05. n.s.=not significant, * $p < 0.05$, ** $p < 0.01$, *** $p < 0.001$. Each point represents an individual animal.

SUPPLEMENTARY DATA

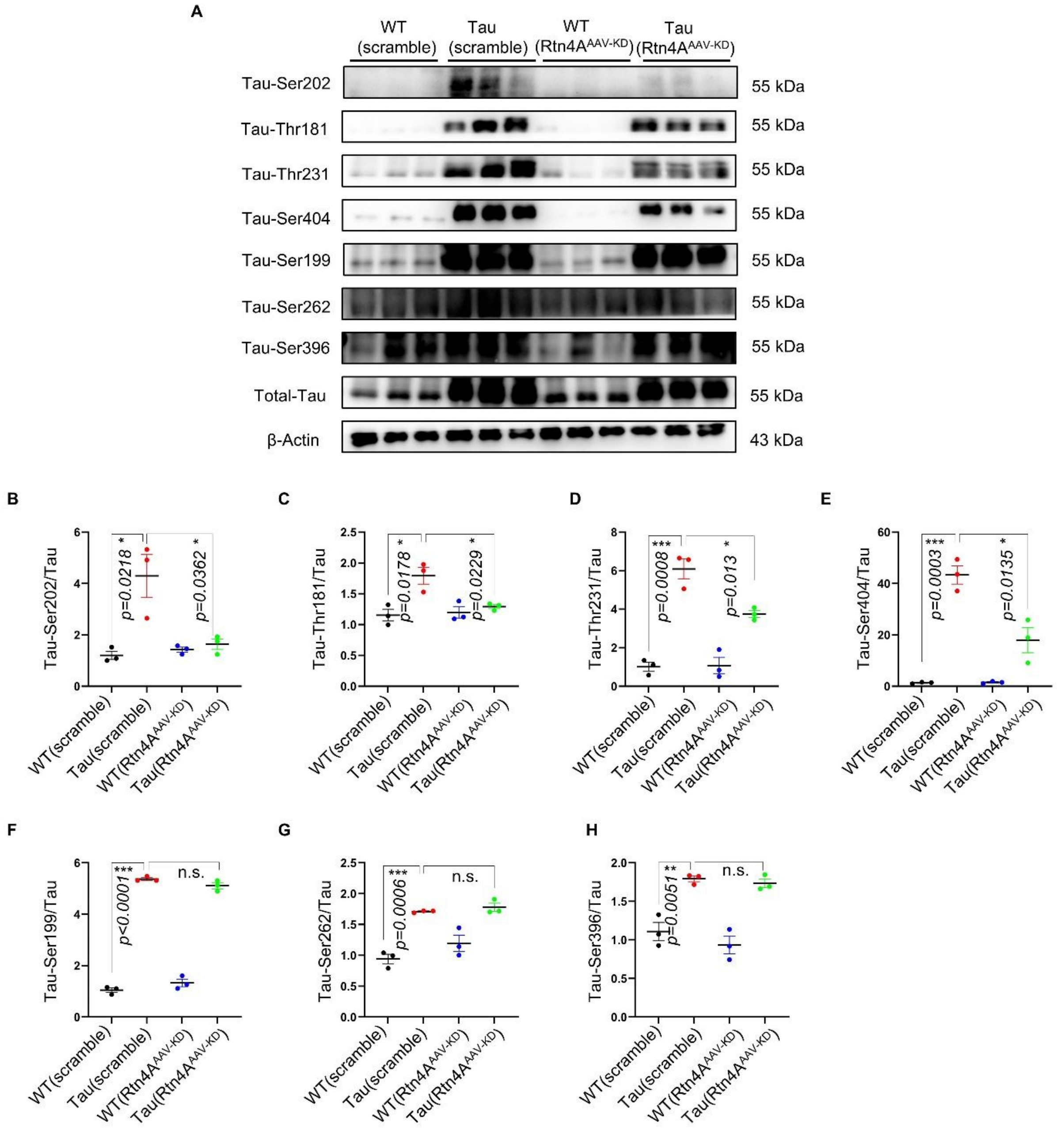

Supplementary Figure 4. Nogo-A knockdown ameliorated tau phosphorylation at multiple sites in hTau. P301S mouse. (**A**) Western blots showing the levels of tau phosphorylation at Thr181, Ser202, Ser199, Thr231, Ser262, Ser396 and Ser404 in the hippocampi of the WT (scramble), Tau (scramble), WT (Rtn4A-AAV-KD) and Tau (Rtn4A-AAV-KD) groups. n=3 in each group. (**B-H**) Data sets were tested for normal Gaussian distribution via Shapiro-Wilk test. Significance was determined by Kruskal-Wallis test, followed by Dunn's multiple comparisons with a significant difference set at 0.05. n.s.=not significant, * $p < 0.05$, ** $p < 0.01$, *** $p < 0.001$. Each point represents an individual animal.

© 2024. Wang Z et al. Published online at http://www.aginganddisease.org/EN/10.14336/AD.2024.0053
